# Treatment of osteoarthritis of the elbow with open or arthroscopic debridement: a narrative review

**DOI:** 10.1186/s12891-018-2318-x

**Published:** 2018-11-10

**Authors:** Keshav Poonit, Xijie Zhou, Bin Zhao, Chao Sun, Chenglun Yao, Feng Zhang, Jingwei Zheng, Hede Yan

**Affiliations:** 1 Department of Orthopedics (Division of Plastic and Hand Surgery), The Second Affiliated Hospital and Yuying Children’s Hospital of Wenzhou Medical University, Key Laboratory of Orthopedics of Zhejiang Province Wenzhou, China, 109 West Xueyuan Road, Lucheng District, Wenzhou, 325027 Zhejiang Province China; 2 Joseph M. Still Burn and Reconstructive Center, 346 Crossgates Blvd, Suite, Brandon, MS 202 USA; 3 Department of Clinical Research Center, The Affiliated Eye Hospital of Wenzhou Medical University, Wenzhou, China

**Keywords:** Elbow, Osteoarthritis, Elbow stiffness, Open, Arthroscopy, Surgery, Debridement

## Abstract

**Background:**

Elbow osteoarthritis (OA) is a common disabling condition because of pain and loss of motion. Open and arthroscopic debridement are the preferred treatment, however there is no consensus on which treatment modality is suited to which category of patient or stage of disease. The objective of this study was to narratively review the literature for a more comprehensive understanding of its treatment options and associated outcomes, trying to provide a better treatment plan.

**Methods:**

The PubMed database, EMBASE, Cochrane Library, and Google Scholar were searched, using the keywords (elbow [title/abstract] and osteoarthritis [title/abstract] and (surgery or open or arthroscop* or debridement or ulnohumeral arthroplasty) including all possible studies with a set of inclusion and exclusion criteria.

**Results:**

A total of 229 studies were identified. Twenty-one articles published between 1994 and 2016 satisfied the inclusion and exclusion criteria including 651 elbows in 639 patients. After comparison, mean postoperative improvement in (ROM) was 28.6° and 23.3°,Mayo elbow performance score/index(MEPS/MEPI) 31 and 26.8 and the total complication rate was 37(11.5%), and 18(5.5%) for open and arthroscopic procedure.

**Conclusions:**

This narrative review could not provide an insight on which surgical procedure is superior to the other due to the poor orthopedics literature. However, from the data we obtained the open and arthroscopic debridement procedures seem to be safe and effective in the treatment of elbow OA. The optimal surgical intervention for the treatment of symptomatic elbow OA should be determined depending on patients’ conditions.

## Background

Arthritis of the elbow is characterized mainly by chronic musculoskeletal pain, stiffness, reduction in the ROM and most importantly a decrease in the quality of life of the patient. Elbow OA has had less focus than lower extremity joints but it can cause severe disability in patients involving their daily living activities [1]. Although the normal range of flexion to extension of the elbow is from 0 degrees to 145 degrees, most daily activities can be accomplished without discomfort within the functional range of 100 degrees (range, 30 degrees-130 degrees) elbow flexion [2]. Nonetheless the elbow provides power for lifting and stability for precision tasks. Consequently, restoration of the normal ROM in a stiff elbow is a major concern [3]. OA is a chronic disorder of synovial joints where there is a progressive disintegration and softening of articular cartilage followed by regeneration of new cartilage and bone at the joint margins (osteophytes), cyst formation and sclerosis in the subchondral bone, mild synovitis and capsular fibrosis contributing to swelling, elbow stiffness and chronic pain. Most patients can go along well with the limitation of ROM, but can’t stand the pain, which severely affects the quality of patients’ life [4].

Numerous procedures have been described in the literature in order to address these symptoms, including arthroscopic soft tissue release, debridement, interposition arthroplasty and total elbow arthroplasty [5–11]. In the recent years, total elbow arthroplasty has shown promising results, but in comparison with hip and knee arthroplasties, a lot more is desired. Due to this reason, procedures like open and arthroscopic debridement have gained popularity and became the mainstay of treatments [12]. In literature, there has been an increased focus on arthroscopic debridement; however there is no consensus on which treatment modality is suited to which category of patient or stage of disease,also there is no objective evidence of clear superiority of any one technique. Each one of the different surgical approaches for elbow OA has its own advantages and disadvantages (see Table 1). The objective of this study was to narratively review the literature for a more comprehensive understanding of its treatment options and associated outcomes, trying to provide a better treatment plan.

**Table 1:** Advantages and disadvantages of Open and Arthroscopic Debridement

Surgery	Advantages	Disadvantages
Open	-Good visualization of joint	-Soft tissue damage
	-Extensive debridement possible	-Larger scars
	-Larger working space	-Risk of soft tissue contraction
	-Most pathologies can be addressed	-Longer rehabilitation
		-Greater risk of infection and hematoma
Arthroscopic	-Minimally invasive	-Tight working space
	-Smaller scars on the skin	-Risk of injury to nerves
	-Less soft tissue damage	-Mainly dependent on surgeon skills
	-Quicker Rehabilitation	-Cannot be used in advanced cases of osteoarthritis due to nerve adhesion

## Methods

**Fig. 1:**
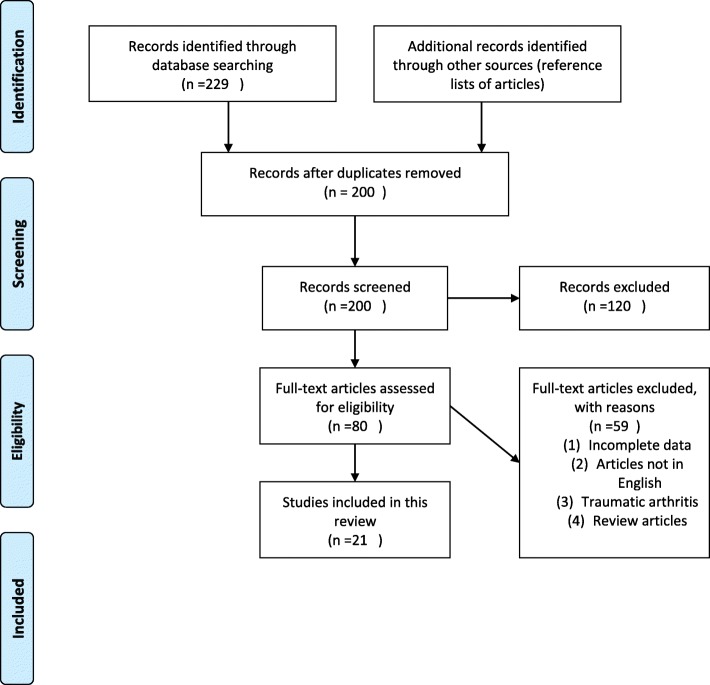
Flowchart showing methodology of review

The PubMed database, EMBASE, Cochrane Library, and Google Scholar were searched for related articles, using the following keywords (elbow [title/abstract] and osteoarthritis[title/abstract] and (surgery or open or arthroscop* or debridement or ulnohumeral arthroplasty) to ensure the inclusion of all possible studies. The search was restricted to articles written in English. In addition, references regarding elbow OA were hand-searched for potential studies. Figure 1 shows the methodology of the review.

### Inclusion and exclusion criteria

To be eligible for inclusion, the studies had to: (1) be published clinical trials; (2) meet the diagnostic criteria for primary elbow OA; (3) report operative treatment of open or arthroscopic and outcomes of elbow OA in humans (4) Articles published in English (5) outcome reported should include at least one of the patient important outcomes (pain, ROM, and functional recovery).

Exclusion criteria were: studies involving Total elbow arthroplasty, elbow replacement, tendon disorders, Fracture of the elbow, interposition arthroplasty, osteochondritis dissicans were excluded.

Each article was thoroughly read and data regarding pre-operative/post-operative flexion, extension, gain in range of motion, complications were retrieved and an average was performed to obtain the gain in range of motion in both groups. Many different scoring methods were used to measure the outcomes of the elbow debridement including (ROM), (MEPS), (MEPI), Andrew and Carson Score, Hospital for Special Surgery (HSS) elbow scoring system, Disabilities of Arm Shoulder and Hand (DASH), Japanese orthopedic association score, Visual Analogue Scale for pain (VAS), Oxford Elbow Score (OES), American Shoulder and Elbow Score (ASES), Quick DASH [12–23]. The most popular scoring system was the ROM and the MEPS which was used in most of the 23 articles. We compared both groups using average gain in range of motion and MEPS but unfortunately the uses of quantitative quality appraisals, quantitative meta-analysis or statistical analysis were not possible because of the limited data available in the literature.. The studies found could not provide solid evidences to aid clinical practice and also the data retrieved were not uniform among all the papers as it was reported by different authors all around the world. We used a narrative literature synthesis approach to summarize the literature, and quantitatively analyzed longitudinal trends across studies where possible.

## Results

**Fig. 2:**
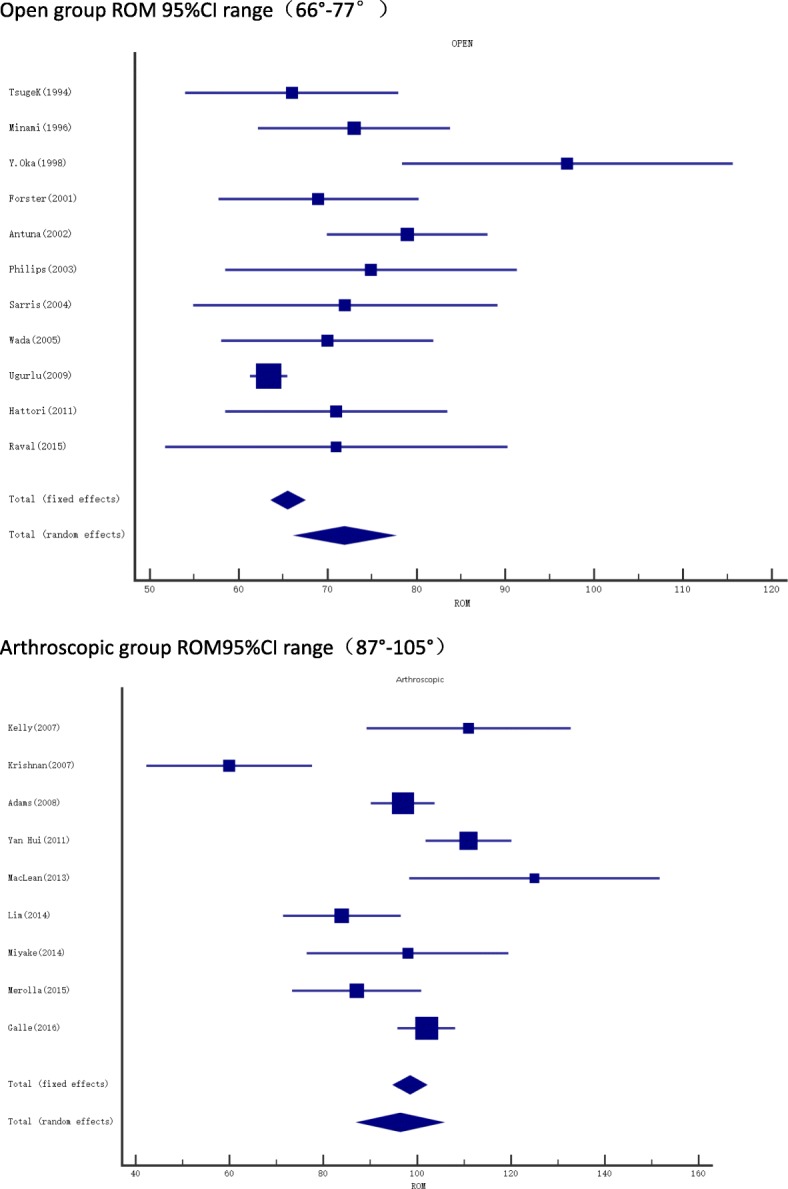
Summary plot for pre-operative ROM, (Data regarding the Pre operative ROM of all the articles in this review was extracted and a summary plot was made. The open procedure shows a lower Pre operative ROM than the Arthroscopic group

A total of 229 studies were identified in the initial search. After a careful review of the lists, full texts were retrieved for 16 articles. The rest of articles that did not meet the inclusion criteria were excluded. A search of the reference lists of selected articles identified 5 more relevant articles; the search was updated with no more relevant articles, leaving a total of 21 articles for the final inclusion. Of the 21 articles 11 were on open joint debridement; 10 were on arthroscopic joint debridement, one study included both open and arthroscopic debridement. These 21 studies included the results of 651 elbows in 639 patients. Of the elbows, 328 were treated arthroscopically, 323 with the open debridement method. Mean improvement in ROM after surgery was 28.6° for Open, 23.3°for Arthroscopic group,mean improvement in MEPS/MEPI after surgery was 31 for Open and 26.8 for Arthroscopic group as shown in Table 2. The difference between the two groups was not significant. All the studies reported good to excellent pain relief after both open and arthroscopic debridement (see Table 3 and Table 4). On the other hand, the pre-operative ROM in the arthroscopic group with an average ROM of 97.2 (87.5–105.3) was much better than that of the open group with an average ROM of 73.3(66.5–77.4). In addition, a significant difference in pre-operative ROM between the two groups was noted in the summary plot, indicating that open procedure is better suited for advanced cases with more limitation of elbow motion (Fig. 2). The total complication rate for the Open procedure was 37(11.5%), and 18(5.5%) for the Arthroscopic procedure as summarized in Table 5. The most common complications for both procedures were ulnar nerve symptoms and hematoma.

**Table 2:** Comparison of open and arthroscopic procedures

	Open	Arthroscopic
ROM (Mean Arc) articles	12	10
Patients	323	328
Improvement	28.6°	23.3°
MEPS/MEPI articles	2	6
Patients	76	203
Pre-Operative Score	57.5	60.74
Post-Operative score	88.5	87.6
Improvement	31	26.8

Abbreviations: Range Of Motion (ROM), Mayo Elbow Performance Score(MEPS)

**Table 3:** Summary of Open Procedures

First Author	Year	Patient Mean Age (year)	Patient Symptoms	Number of patients and elbow (n=)	Type of treatment	Follow Up (Months) Range/mean	Evaluation Methods	Pain scale	Treatment Outcomes (Mean ROM)
Tsuge K et al [19]	1994	59	Pain and loss of range of motion	28 patients, 29 elbows	Open joint debridement	64	System of Japanese Orthopedic association	Good pain relief. No measure reported	Improvement of 33.2°
Minami et al[27]	1996	48.6	Pain on terminal motion, loss of range of motion	44 patients, 44 elbows	Outerbridge Kashiwagi procedure	127	(ROM)	27 out of 44 reported good pain relief. No measure reported	Improvement by 17°
Y.Oka[28]	1998	32	Severe pain at terminal flexion and extension, loss of range of motion	26 patients, 26 elbows	Open procedure (lateral and medial approach) and Outerbridge Kashiwagi procedure	46	ROM	0–2 grading scale (before 2 to after 0.24)	Improvement of 24°
Cohen et al[14]	2000	55	Pain, Stiffness, Locking	16 Patients, 18 Elbows	O-K Procedure	35.3	ROM and pain score	0–6 Likert scale from MEPI (after 2 points)	Improvement of 15°
Forster et al[20]	2001	55	Pain, decrease in range of motion, locking	35 patients, 36 elbows	Outerbridge Kashiwagi pr0cedure	39	ROM, pain score	Morrey’s system (0–3 Likert scale). (1.8 before to 1.1 after)	(PRE OP39°-108°) (POST OP27°-121°)
Antuna et al[21]	2002	48	Pain with terminal elbow extension	45patients, 46 elbows	Open ulnohumeral arthroplasty	80	MEPS	76% had complete pain relief. No measure reported	Improvement of 22°
Philips et al[29]	2003	51.4	Pain and loss of flexion/extension	19 patients, 20 elbows	O-K procedure	75	(DASH) and (MEPS)	All patients reported pain relief. No measure reported.	Improvement of 20°
Sarris et al[30]	2004	52	patients was pain in terminal flexion and extension	17 patients, 17 elbows	Outerbridge Kashiwagi procedure	36	Pain scale, ROM	Morrey’s system (0–3 Likert scale). All patient 0 post operatively.	PRE OP(26° to 98°) POST OP(14° to 118°)
Wada et al[32]	2005	50	Loss of range of motion,pain	32 patients 33 elbows	Debridement arthroplasty	121	ROM	Improved from 13 to 27	Improvement of 24°
Ugurlu et al[22]	2009	47	Pain and loss of range of motion	10 patients 10 elbows	Ulnohumeral arthroplasty	25 to 46	Andrews and Carson score	VAS (before 8; after 3.1)	flexion-extension arc improved from 63.4° to 120°
Hattori et al[23]	2011	59	Pain at the end points of motion	31 patients 31 elbows	Debridement arthroplasty combined with capsulectomy	19 ± 7	(MEPS)	23 painful, 8 mildly painful post operatively. No measure reported.	mean arc of elbow motion Increased by 40° +/_ 13°.
Raval et al[12]	2015	54	Pain and stiffness at the extremes of movements	13 Patients 13 elbows	Ulnohumeral arthroplasty	48	(Quick DASH), (VAS)	VAS (before 8 after 2)	Improvement of 27 degrees in the flexion extension arc

Abbreviations: Range Of Motion(*ROM*), Mayo Elbow Performance Score(*MEPS*), Disabilities of Arm Shoulder and Hand(*DASH*), American Shoulder and Elbow Score(*ASES*), Visual Analogue Scale(*VAS*), Oxford Elbow Score(*OES*), Mayo Elbow Performance Index(*MEPI*), Hospital for Special Surgery (*HSS*) elbow scoring system**。**

**Table 4:** Summary of Arthroscopic Procedures

Author	Year	Patient Mean Age (years)	Patient Symptoms	Number of patients and elbow (n=)	Type of treatment	Follow Up (Months) Range/mean	Evaluation Methods	Pain scale	Treatment Outcomes (average Flexion/extension arc)
Cohen et al. [14]	2000	46	Pain, Stiffness, Locking	26 Patients, 26Elbows	Arthroscopic debridement	35.3	ROM and pain score	0–6 Likert scale from MEPI (after 2.9 points)	Improvement of 18°
Kelly et al. [33]	2007	51	Pain and loss of range of motion	24 patients 25 elbows	Arthroscopic debridement	24 to 123	Andrews and Carson score	Decreased from 7 to 2	Improved by 21°
Krishnan et al. [15]	2007	36	Pain and loss of range of motion	11 patients 11 elbows	Arthroscopic ulnohumeral arthroplasty	24–29	(VAS),(MEPS)	Decreased from 9.2 to 1.7	improvement of 73°
Adams et al. [37]	2008	52.8	Pain and loss of range of motion	50 patients 52 elbows	Arthroscopic debridement	26–68	ROM,MEPI	Subjective pain(0–5) Decreased 2.86 to 1.44	Improvement of 26.23°
Yan Hui et al. [16]	2011	23 ± 5	Pain, Locking, Loss of Range of motion	35 Patients 36 elbows	Arthroscopic debridement	16–98	(HSS), ROM,pain scale	All atheletes reported pain improvement.	Improved by 16°
MacLean et al. [17]	2013	42	Pain and locking	20 patients 21 elbows	arthroscopic debridement	66	(DASH), Mayo, and ROM	Measure not reported.	unchanged
Lim et al.(8)	2014	51.4	Terminal pain at flexion and extension with limitation of motion	43 patients 43 elbows	Arthroscopic d ebridement	38	(VAS),(MEPI)	Decreased from 4.5 to 2.2	mean flexion improved from 103° to 116°
Miyake et al. [34]	2014	38	Pain at the endpoints of movement and stiffness, catching or locking	20 patients 20 elbows	Arthroscopic debridement	24 to 29	Mayo Elbow Performance Score, Range of motion	No measure reported, pain disappeared or decreased post operatively.	Flexion PRE OP from 121° to to 130° POST OP
Merolla et al. [35]	2015	48	Pain, limited range of motion	48 patients 48 elbows	Arthroscopic joint debridement	44	(ROM), pain score, (OES), and (MEPS)	Decreased from 7.2 ± 1.6 to 4.3 ± 1.1	,Flexion PRE OP from 115.73° ± 16.53° to128.75° ± 12.35° POST OP
Galle et al. [36]	2016	48	Loss of elbow motion, Pain	46 patients 46 elbows	Arthroscopic osteo-capsular arthroplasty	40.8	(VAS), (MEPS), (DASH), (ASES)	ASES pain Score post op 40 +/−12	flexion (PRE OP from 126° to 135° POST OP

Abbreviations: Range Of Motion(*ROM*), Mayo Elbow Performance Score(*MEPS*), Disabilities of Arm Shoulder and Hand(*DASH*), American Shoulder and Elbow Score(*ASES*), Visual Analogue Scale(*VAS*), Oxford Elbow Score(*OES*), Mayo Elbow Performance Index(*MEPI*), Hospital for Special Surgery (*HSS*) elbow scoring system

**Table 5:** Complications of open and arthroscopic debridement (n = number of elbows)

Complications	Open (n=)	Arthroscopic (n=)
	323	328
superficial wound infection	3	2
shoulder-hand syndrome	1	–
deep wound infection	1	1
ulnar nerve symptoms	27	6
Radial nerve palsy	1	–
residual loose bodies	–	2
hematoma	4	5
Recurrent effusions	–	2
Total	37(11.5%)	18(5.5%)

## Discussion

The etiology of primary OA of the elbow has not been fully elucidated. Several studies have proposed environmental factors as the primary etiology. No known morphologic features of the elbow have been identified as a predisposition to the development of primary OA of the elbow [24].The cardinal features of OA are (1) progressive cartilage destruction, (2) subarticular cyst formation, (3) sclerosis of surrounding bone (4) osteophyte formation (5) capsular fibrosis. Patients complain of pain and stiffness at the extremes of movements. This can be caused by degenerative changes at the radio-capitellar, ulnohumeral joint or due to ulnar nerve symptoms [5].

Non-operative management which includes elbow sleeves, non-steroidal anti-inflammatory medications, and intra-articular corticosteroid injections remains the mainstay of initial treatment for both primary OA of the elbow and posttraumatic arthritis of the elbow [3, 25, 26]. In the management of primary OA of the elbow, surgical interventions are used when conservative measures like physiotherapy and medical management fail. The most common indications for surgery are end range pain, stiffness, loose bodies, and locking of the elbow joint. Open and arhroscopic debridement are the preferred surgical management. In terms of these two procedures nearly all the studies in this review reported excellent reduction of pain, substantial increase in ROM, and functional recovery, however, a lack of trials limits the ability to directly compare different treatment options. All the studies included did not use the same scoring system and as a result direct comparison was not always possible. However, ROM and MEPS were used in some studies and therefore a direct comparison was made possible and the results of were as follows: Mean improvement in ROM after surgery was 28.6° for Open, 23.3°for Arthroscopic group,mean improvement in MEPS/MEPI after surgery was 31 for Open and 26.8 for Arthroscopic group. Both groups had similar outcomes in terms of improvement therefore it shows that both open and arthroscopic procedures still have its role in the treatment of osteoarthritis.

In this study we found that by comparing the open [12, 14, 19–23, 27–32] and the arthroscopic [13–18, 33–37] procedures, both procedures have comparable outcomes concerning ROM and MEPS. This proves that both open and arthroscopic procedure can improve function, ROM and relieve pain in patients with elbow OA. Pain is the major complaint for patients resorting to surgery. In this review most studies reported good to excellent pain relief after open joint debridement. However, to evaluate pain different scores were used, including (VAS) [3, 25], Morrey’s 0–3 Likert scale pain grading system [6, 26], but other studies [8, 14] despite achieving satisfactory relief in pain did not mention the outcome measure they used. Similarly after arthroscopic debridement, every author reported excellent pain relief. The VAS was adopted in five studies and significant changes in pain levels were reported [15–19]. Krishnan et al. [15] followed 11 elbows and mentioned a change in the mean VAS scores from 9.2 Pre-surgery to 1.43 after final follow up at 26 months. A Likert scale from 0 to 5 was used in one study [11]. Others studies used Morrey’s scoring system, elbow scoring systems like MEPI and good amelioration in pain levels were reported. In terms of complications the open group had a higher rate (11.5%) than the arthroscopic group (5.5%). Some of the complications reported were superficial wound infection, shoulder-hand syndrome, deep wound infection, ulnar nerve symptoms, Radial nerve palsy, residual loose bodies, hematoma and recurrent effusions. The two most common complications in both groups were ulnar nerve symptoms and hematoma formation. Open debridement requires an extensive soft tissue dissection, division of collateral ligament whereas arthroscopic debridement requires small stab incision and is minimally invasive; it requires expertise and specialized equipment’s. This may explain why open group had a higher complication rate than the arthroscopic group but there is also the fact that open group had lower preoperative ROM which means that the patients were in a much advanced stage of osteoarthritis than the arthroscopic group, this might also contribute to a higher complication rate in the open group.

Cohen et al. [14] compared open joint debridement (18 elbows) to arthroscopic debridement (26 elbows) in 44 elbows. After a total follow up of 3 years they found a greater mean increase in flexion-extension arc of 21 degrees in the open debridement group, and a mean increase of 7 degrees in the arthroscopic debridement group. Although the authors reported superior pain relief with the arthroscopic procedure, a greater improvement in flexion was noted with the standard open procedure. This finding is not surprising as anterior capsular contractures are much more amenable to arthroscopic release than are contractures involving the posterior structures. Because the posterior bundle of the medial collateral ligament contracts and prevents flexion in patients with a long-standing lack of flexion, gains in extension are greater after arthroscopic release [4]. Therefore, more studies comparing different treatment options are needed to be able to gain insight on which procedure is superior to the other.

Open and Arthroscopic Procedure also proved to be safe in athletes’,and a younger generation of patients. Two studies performed debridement in professional athletes’ with one using open procedure and the other using the arthroscopic procedure. Oka et al. [28] treated twenty-six elbows in 26 patients using open debridement, preoperatively all patients complained of pain level around grade 2 or grade 3. There was limited ROM with a mean lack of extension of 16° (range 0°–30°), and a mean flexion range of 113° (range 80°–140°). In all cases severe pain occurred at the end of flexion or extension. As a result, anxiety and a decrease in performance while performing physical activities were noted. The concerned sports included baseball and Judo each affecting (9 cases), aikido (3 cases), apparatus gymnastics (2 cases), and karate, volleyball and bodybuilding (one case each). The mean sport participation time was 12 (3–30) years. Surgery resulted in improved pain relief and ROM. However, there was one case that suffered residual pain rated as grade 2. Each and every athlete returned to their prior activities and first class accomplishments was achieved by a few. Despite a minor recurrence of osteophytes, long-term results also indicated that the improvement in pain and ROM was maintained over a prolonged period.

Yan et al. [16] used the arthroscopic procedure to treat 35 professional athletes. The concerned sports included wrestling affecting 8 elbows in 7 athletes, weightlifting and judo both affecting 5 cases each, shooting, boxing, diving, ping-pong, and rowing (one case each), and badminton (3 cases), gymnastics, javelin, softball, basketball and baseball (2 cases each). All the 35 patients complained of pain. Before surgery, mean total arc of motion was (111 ± 28) ° (range 50°–150°), mean loss of extension was (14 ± 12) ° (range 0° – 40°), and mean flexion was (125 ± 20) ° (range 75°–150°). The mean sport participation time was (8 ± 6) years (range 2–20 years). Before surgery all patients reported difficulties performing physical activity: slightly affected in 2 (6%) elbows, severely affected in 24 (67%) and unable to participate in 10 (28%). Postoperatively as per the HSS scoring system, the outcome was as follows: poor for 6, good for 14 and excellent for 16 elbows. Pain improvements were reported by all athletes. After debridement, mean total arc of motion was 127° ± 26° (range 80°–150°), mean extension loss was 7° ± 12° (0°–30°), and mean flexion was 134° ± 17° (95°–150°). Post-surgery total arc of motion increased 16°, extension increased 7° and flexion increased 9°.

**Fig. 3:**
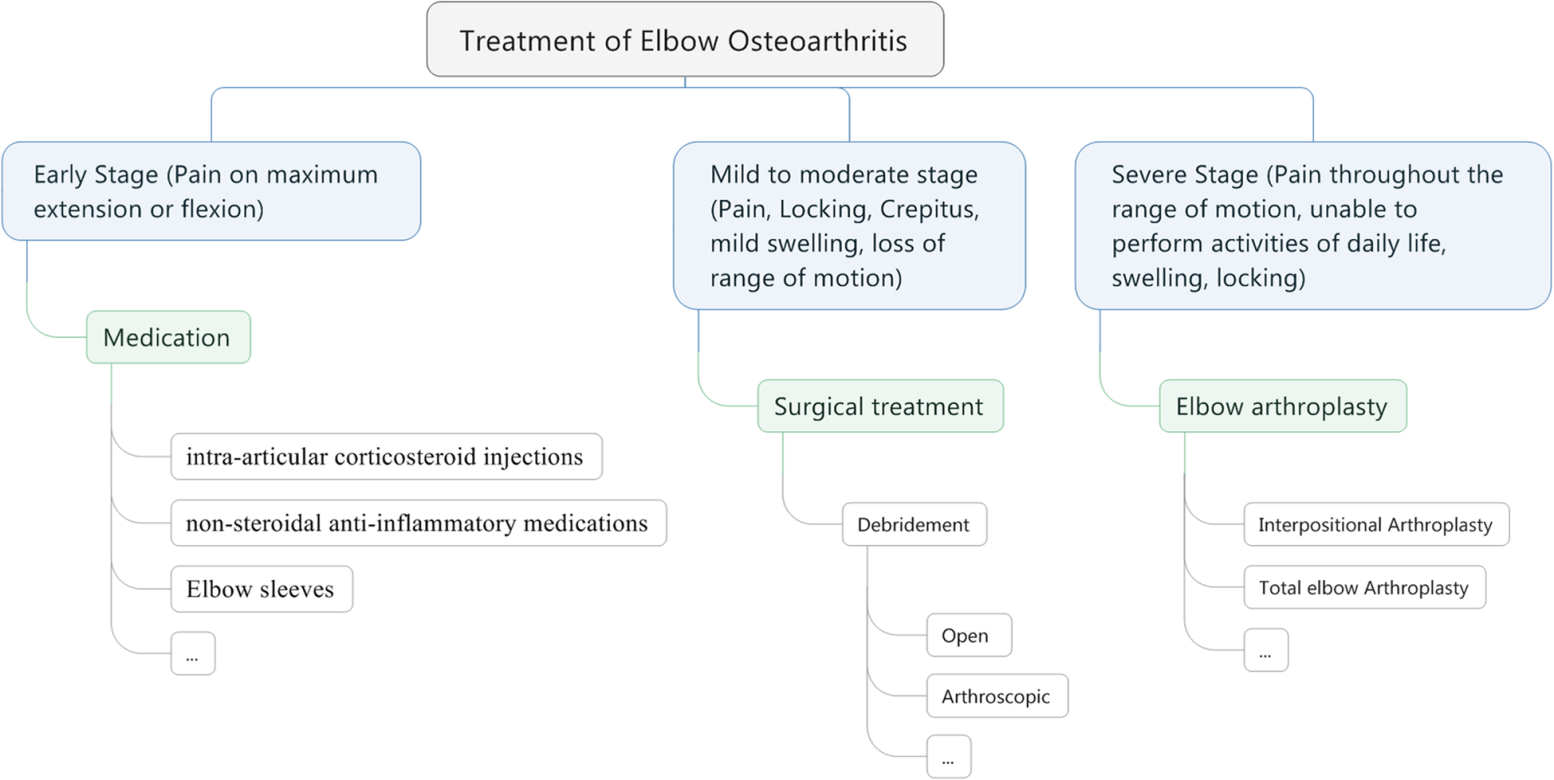
Treatment strategy for elbow OA (Describing the treatment for early, mild, moderate and severe stage of osteoarthritis)

A basic treatment strategy was proposed according to our findings from studies selected in this narrative review as shown in Fig. 3. We have detailed the treatment of osteoarthritis and classified it into early stage, mild to moderate stage, severe stage and further explained which treatment is more appropriate for each stage and the severity/type of pain for each stage has been described. This treatment strategy will benefit the surgeon to identity the stage of osteoarthritis and have a general idea about the treatment needed for that stage, furthermore open treatment seems to have its place mostly in rural institutions where there are less facilities available and the surgeons doesn’t require much expertise to perform the operation, whereas arthroscopic treatment is becoming popular in urban institutions but it requires a greater learning curve and is more challenging than open technique. People in urban areas want a smaller scar and a quicker recovery and tend to be able to afford the high cost of the operation. The orthopedic literature is poor and the studies used in this narrative review are case series which prevent us to draw a valid conclusion, this calls for more Randomized controlled trials comparing different surgical options either in terms of short- or long-term follow-up that will enable us to better compare these two procedures.

Recently, arthroscopy has gained popularity in clinical practice and we found that similar outcomes were achieved by both open and arthroscopic procedures, but the complication rate was lower in the arthroscopic group. However differences in pre-operative ROM was significantly different, the results of the summary plot showed a better pre-operative ROM in the arthroscopic group compared to the open group, indicating that open procedure is better suited for advanced cases and the arthroscopic procedure may be more acceptable for mild to moderate cases.

This narrative review had limitations as there is a scarcity of data on comparative outcomes and a lack of prospective studies that directly compare the two surgical techniques. The most noticeable finding was a lack of Randomized controlled trials that bear comparison with short- or long-term benefit among different debridement procedures. However, reviews that analyze and summarize these procedures could boost our confidence about joint debridement procedures being safe and efficacious. The uses of quantitative quality appraisals, quantitative meta-analysis or statistical analysis were not possible because of the limited data available in the literature. Furthermore, there were many scoring methods used to measure the outcomes of the elbow debridement. Researchers used more than 1 score, but direct comparison of all results was not possible because of this heterogeneity. The vast variety of scores calls for a widely acceptable questionnaire that will give us the possibility to measure the wide range of patients. As a matter of fact, we also noticed that patients in the open surgery group seemed to suffer from a more severe OA with low pre-operative ROM than those in the arthroscopic group, which may definitely cause selection bias for analysis. To our knowledge this is the first review that compares results across the two most common debridement procedures and this can help the general audience to better understand the outcomes, benefits and complications of these two procedures.

## Conclusion

The optimal surgical procedure for the treatment of OA of the elbow is controversial. This narrative review could provide an insight on which surgical procedure is superior to the other due scarcity of data from the literature. However, from the data we obtained the open and arthroscopic debridement procedures seem to be safe and effective in the treatment of elbow OA. The optimal surgical intervention for the treatment of symptomatic elbow OA should be determined depending on patients’ conditions. A clear understanding and knowledge of each approach will help the surgeon to evaluate the most appropriate approach for any given surgery. Reviews and case series directly comparing the two approaches and longer-term follow-up are the key to clearly elucidate the respective roles of these two surgical approaches.

## Abbreviation

ASES: American Shoulder and Elbow Score
DASH: Disabilities of Arm Shoulder and Hand
HSS: Andrew and Carson Score, Hospital for Special Surgery elbow scoring system
MEPS/MEPI: Mayo elbow performance score/index
OA: Osteoarthritis
OES: Oxford Elbow Score
ROM: Range of motion
VAS: Visual Analogue Scale for pain,
